# Optimization of magnetic reference layer for neutron reflectometry

**DOI:** 10.1107/S1600576725004674

**Published:** 2025-07-22

**Authors:** Anton Zubayer, Fredrik Eriksson, Naureen Ghafoor, Jochen Stahn, Jens Birch, Artur Glavic

**Affiliations:** a Department of Physics, Chemistry and Biology (IFM) Linköping University Olaus Magnus väg 37 Linköping58431 Sweden; b PSI Center for Neutron and Muon Sciences, Forschungsstrasse 111, Villigen PSI5232, Switzerland; Oak Ridge National Laboratory, USA; North Carolina State University, USA

**Keywords:** magnetic reference layers, thin films, reflectometry, neutron scattering, sensitivity

## Abstract

Neutron reflectivity is a powerful technique for probing density profiles in films, with applications across physics, chemistry and biology. However, challenges arise when dealing with samples characterized by high roughness, unknown scattering length density (SLD) with low contrast, very thin layers or complex multi-layered structures that cannot be uniquely resolved due to the phase problem. Incorporating a magnetic reference layer (MRL) and using polarized neutron reflectivity improves the sensitivity and modelling accuracy by providing complementary information. In this study, we introduce a quantitative means of comparing MRL systems in a model-free way. We apply this approach to demonstrate that CoTi alloys offer a superior solution as MRLs compared with the commonly used Fe or Ni MRLs. The low nuclear and magnetic scattering length densities of CoTi significantly enhance sensitivity, making it particularly advantageous for soft-matter research. Furthermore, the tunable Co versus Ti ratio allows for optimization of the SLD to achieve maximum sensitivity, establishing CoTi as a highly effective choice for MRL applications. The applied simulation framework for optimizing MRL sensitivity to a specific materials system and research question is a generic approach that can be used prior to growing the MRL for a given experiment.

## Abstract 2

This study introduces a quantitative framework for evaluating magnetic reference layers (MRLs) in polarized neutron reflectometry and demonstrates that CoTi alloys outperform conventional Fe- and Ni-based MRLs. The tunability of CoTi’s nuclear and magnetic scattering length densities enables superior sensitivity, making it a highly effective choice for enhancing contrast and resolving phase ambiguity in soft-matter and thin-film research.

## 1. Introduction

Neutron reflectometry (NR) is a versatile technique for analysing the depth profile of thin films and layered materials, *i.e.* their thickness, density and roughness. It is particularly impactful in soft-matter research, where it probes complex systems like polymers, lipids and biological macromolecules under near-natural conditions (Charlton *et al.*, 2011 ▸; Yuan *et al.*, 2011 ▸; Gerstenberg *et al.*, 1996 ▸; Skoda, 2019 ▸; Steyerl, 1991 ▸). The ability of NR to investigate interfaces and thin films in soft materials reveals crucial insights into phenomena such as polymer dynamics, protein adsorption and lipid membrane organization (Yuan *et al.*, 2011 ▸; Torikai *et al.*, 2007 ▸; Dadmun, 2015 ▸; Fragneto & Menelle, 2011 ▸). Its sensitivity to water is also key for studying hydration effects in biological and soft-matter contexts, making NR essential for advances in materials science and biotechnology. However, when it comes to studying non-magnetic layers with unknown scattering length density (SLD) and morphology, traditional NR encounters significant challenges due to the phase problem (Fiedeldey *et al.*, 1992 ▸; Farhad Masoudi & Pazirandeh, 2005 ▸; Kasper *et al.*, 1998 ▸). A low contrast between layer and substrate results in weak oscillations and leads to reduced sensitivity in the data analysis. Missing knowledge about the expected layer morphology and SLD profile can yield different models reproducing the measurements quite well but with vastly different physical interpretations (Sivia & Pynn, 1992 ▸; Kaiser *et al.*, 1994 ▸; Majkrzak *et al.*, 2003 ▸). In experiments where one component of the sample is submerged in water, this problem is often tackled by measuring different H_2_O/D_2_O mixtures, leading to contrast variation. While this technique is proven it cannot always be applied, as a change in contrast, for example, might require the creation of a new sample that could have a different SLD profile.

The introduction of magnetic reference layers (MRLs) and the use of polarized neutron reflectivity (PNR) measurements address these challenges in a similar way. By exploiting the distinct SLD contrasts for spin-up and spin-down neutrons, the use of an MRL then allows for the generation of two separate reflectivity curves. This dual-curve approach can lift ambiguity and help to overcome the phase problem, significantly improving the sensitivity and thus the reliability of data interpretation. It effectively circumvents the ambiguity associated with weak contrast by the fact that at least one of the spin states cannot have the same SLD as the substrate. Thus, while NR is quicker and more accessible, PNR with MRLs offers the capability to reveal features that NR might overlook. It also provides greater reliability due to its higher sensitivity and reduced risk of misinterpretation.

Organic thin films typically exhibit low nuclear SLDs, often in the range of 0 to 4 × 10^−6^ Å^−2^ (Yepuri *et al.*, 2016 ▸; Murphy *et al.*, 1999 ▸; Yu *et al.*, 2020 ▸), making NR highly dependent on the careful selection of contrast layers. To enhance magnetic sensitivity in PNR, MRLs are introduced, but their effectiveness is strongly influenced by their own spin-dependent SLDs relative to that of the sample/layer of interest (SOI).

A widely used MRL is iron (Fe), which has a nuclear SLD of approximately 8 × 10^−6^ Å^−2^ and a magnetic SLD of about 5 × 10^−6^ Å^−2^. This results in a spin-up SLD of approximately 13 × 10^−6^ Å^−2^ and a spin-down SLD of around 3 × 10^−6^ Å^−2^ (Sears, 1992 ▸). Although this spin contrast may seem beneficial at first, the very high spin-up SLD leads to dominant neutron reflection from the MRL itself, often overshadowing the reflectivity signal from the SOI, especially when the SOI has a low SLD, typical of organic materials. This effect is not unique to Fe; similar limitations are observed in other high-SLD MRLs such as Ni. Therefore, to preserve sensitivity to the SOI, it is essential to minimize the SLD of the MRL in both spin-up and spin-down states.

To address this, the nuclear and magnetic SLDs of the MRL can be tuned, as suggested in our prior work (Zubayer *et al.*, 2024 ▸). Among ferromagnetic elements, Co is advantageous due to its relatively low nuclear SLD of 2.3 × 10^−6^ Å^−2^. However, for optimal performance, even lower SLDs are preferable, ideally keeping the spin-up SLD below that of the substrate and the SOI. This can be achieved by alloying Co with Ti, where Ti has a negative nuclear SLD (−1.9 × 10^−6^ Å^−2^), to form a CoTi alloy. In addition to reducing the nuclear SLD, this dilution also decreases the magnetic SLD, thereby suppressing the spin-up SLD further and improving sensitivity to low-SLD SOIs.

In this study, we introduce a quantitative means of comparing MRL systems in a model-free way. The samples in this study represent a range of typical thin-film layers encountered in soft matter and related fields, with specific variations in thickness, roughness and SLD. Note that in this study SOI refers only to the layer of interest and not the entire sample put into the beam. The SLD of the SOI can vary between 1 × 10^−6^ Å^−2^ and 3 × 10^−6^ Å^−2^, reflecting the diverse range of soft-matter compositions, from low-density polymers to denser organic materials. To capture these variations, this study uses a matrix of nine different SOIs, combining three structural types (normal, rough and thin) with three different SLD values (1, 2 and 3 × 10^−6^ Å^−2^). A ‘normal’ SOI is modelled as a 500 Å thick layer, which is representative of many soft-matter systems such as polymer films, lipid bilayers, protein-coated surfaces or perovskite layers (Kühner *et al.*, 1994 ▸; Shahal *et al.*, 2008 ▸; Shoaib *et al.*, 2017 ▸; Momblona *et al.*, 2014 ▸). These materials often fall within this thickness range due to their structural stability and relevance in applications like membranes or thin coatings. The ‘rough’ SOI maintains the same 500 Å thickness but features an increased roughness of 50 Å instead of the typical 15 Å. Conversely, some samples, especially in soft matter, can be very thin; our ‘thin’ SOI represents such cases with a thickness of 50 Å and a moderate roughness of 15 Å, mimicking self-assembled monolayers, thin polymer brushes or confined lipid bilayers (Hausch *et al.*, 1998 ▸; Zeineldin *et al.*, 2006 ▸). This approach allows for a comprehensive investigation of how MRLs perform in terms of sensitivity to these diverse SOIs. The MRLs studied include a scenario with no MRL, an Fe MRL, an Ni MRL and a novel CoTi MRL. These configurations are evaluated for their effectiveness in distinguishing the SOI’s structural and magnetic properties. By systematically exploring the sensitivity of these MRLs across the nine SOIs, this paper provides insights into how well each MRL configuration performs under varying experimental conditions, highlighting the potential advantages of the CoTi MRL in delivering robust and reliable results for soft-matter systems. Finally, a two-layer SOI is used to extrapolate the findings on more complex and realistic SOIs to corroborate the advantage of CoTi as an MRL.

**Figure 1:**
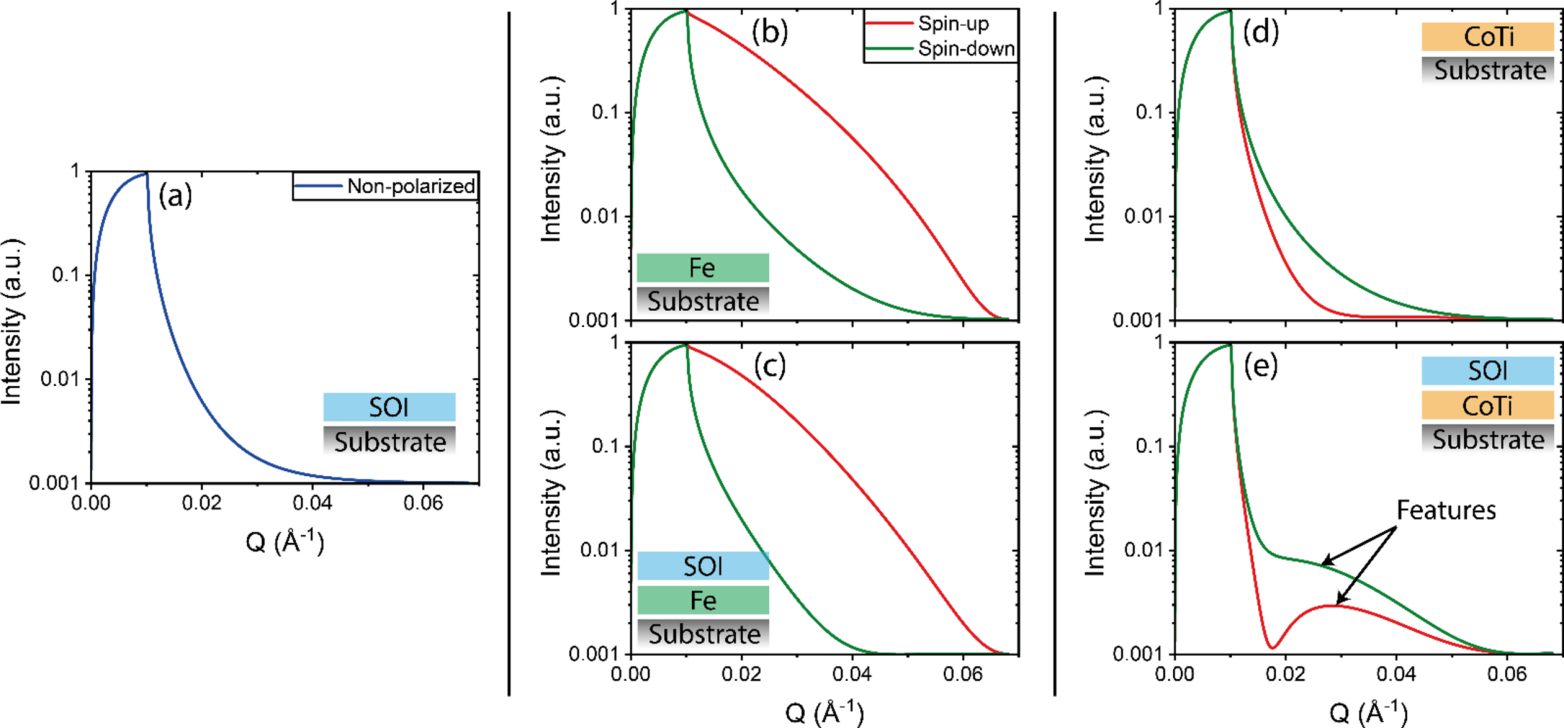
Polarized neutron reflectivity simulations of five cases. (*a*) Sample of interest layer with an SLD of 2 × 10^−6^ Å^−2^ on substrate, (*b*) Fe layer on substrate, (*c*) SOI on Fe on substrate, (*d*) Co_0.73_Ti_0.27_ layer on substrate and (*e*) SOI on Co_0.73_Ti_0.27_ on substrate. The substrate is Si for all samples. The simulations include a beam footprint and a background of 0.001. *Q* is the reciprocal-space vector.

As seen in Fig. 1 ▸, NR from only the SOI shows no features, while when using an Fe MRL it is hard to distinguish between only the MRL and the MRL with the SOI. The suggested CoTi MRL and MRL with SOI show a clear difference, as well as showing evident features of the SOI.

The figures of merit (FOMs) for PNR that we have developed quantify the effectiveness of MRLs in distinguishing spin-polarized interactions with SOIs. The sensitivity FOM (SFM) integrates the absolute sensitivity across the momentum transfer range, accounting for both spin-up and spin-down neutron channels. The magnetic contrast FOM (MCF) measures the difference between spin states, representing the information gain. Finally, the total sensitivity figure (TSF) combines the contributions from all sensitivity metrics across multiple SOI types, providing a comprehensive evaluation of the MRL’s performance. Details can be found in the *Methods*[Sec sec2] section and in the supporting information.

In this study, we use simulations to characterize the efficacy of MRLs and of the proposed CoTi MRLs against Fe or Ni MRLs, and we use experimental results to find the optimal ratio between Co and Ti for the best possible performance of an MRL.

## 2. Methods

The *GenX3* software (Glavic & Björck, 2022 ▸) was employed for conducting PNR simulations to analyse the reflectivity responses for both spin-up and spin-down neutrons across the samples listed in Table 1 ▸. *GenX3* was also utilized to fit the data collected from PNR measurements. This software uses the Parratt recursion formalism, which recursively simulates and fits reflectivity data by considering each interface to compute the overall detected intensity. The fitting results generated by the software were instrumental in determining parameters such as thicknesses, roughness, and both nuclear and magnetic SLDs. Lastly, *GenX3* was used to simulate the sensitivities of Fe, Ni and CoTi MRLs. A custom Python script was developed within *GenX* to calculate reflectivity and sensitivity curves for various MRLs and SOIs. Details of the simulations can be found in the supporting information and the code and files are available at https://github.com/Azubayer/GenX-sensitivity.

Table 2 ▸ shows the MRLs used for the reflectivity, SLD depth profiles and sensitivity simulations.

Specular reflectivity 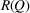 represents the fraction of neutrons reflected from the sample as a function of the momentum transfer 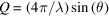, where λ is the neutron wavelength and θ is the angle of incidence (Felcher, 1989 ▸). A critical factor in this process is the SLD, which serves as a fundamental parameter describing the interaction of neutrons with a material (Teubner, 1991 ▸). The reflectivity is influenced by the variation in the SLD across interfaces. Reflectivity is calculated using the matrix formalism, which incorporates the nuclear [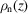] and magnetic [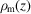] SLD profiles throughout the sample structure. The algorithm also accounts for interference effects between neutron waves reflected at different interfaces within the multilayer structure, producing characteristic oscillations in the reflectivity 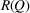. Additionally, the impact of interface roughness is considered, as it introduces Gaussian damping, reducing the reflectivity intensity at higher *Q* values.

By modelling the reflectivity across various candidate reference layers, their performance in extracting information about the SOI over a specific *Q* range can be assessed, enabling MRL optimization for specific PNR experiments. In PNR, the spin-dependent SLD can be calculated from the nuclear and magnetic contributions,
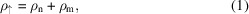

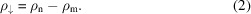
The nuclear SLD 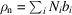, where *N* is the atomic number density and *b* is the coherent scattering length, and the magnetic SLD (Felcher *et al.*, 1986 ▸; Li *et al.*, 2021 ▸) 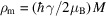, where *M* represents the layer magnetization. Note that the definition of 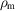 is true if there are no spatial variations in the magnetic field. For an optimal magnetic reference layer, the magnetic SLD 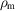 must provide sufficient contrast with the nuclear SLDs of the adjacent layers. This contrast ensures that the spin polarization dependence of the reflectivity remains detectable over a broad *Q* range, enabling accurate characterization of SOI structures. The reflectivity in PNR experiments is measured for neutrons in two distinct polarization states: spin-up 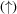 and spin-down 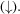 These states interact differently with the magnetic field of the reference layer, leading to spin-dependent reflectivity curves.

By analysing these curves, we can assess how well an SOI is measured using a reference layer. We define the sensitivity 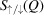 to quantify how well an experiment can distinguish between the reference substrate and the additional SOI. It is defined for the spin-up and spin-down channels as
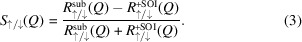

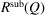
 represents the reflectivity for the empty MRL substrate, while 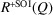 corresponds to the reflectivity after the SOI is added to this substrate. Plotting the sensitivity allows visualization of the effect of the SOI on the reflectivity curve and makes it possible to gauge the information gained with the magnetic contrast. An ideal reference layer will exhibit a strong and consistent sensitivity over a broad *Q* range to probe the SOI properly, as well as a large difference based on the spin-state for maximum information gain to resolve the phase problem.

To evaluate the global sensitivity of the MRL across the momentum transfer range, we define three FOMs, as mentioned in the previous section. The sensitivity figure of merit 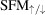 quantifies the total area between the 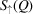 or 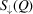 curve and the horizontal line *S* = 0 over the momentum transfer range *Q*_min_ to *Q*_max_. By taking the absolute value 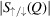, both positive and negative changes for the SOI are considered. The integration bounds *Q*_min_ and *Q*_max_ define the measured *Q* range over which the analysis is conducted. The additional magnetic contrast figure (MCF) quantifies the difference between the spin states, which is a measure of the information gain from the two independent channels:
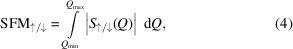

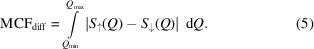
Finally, the total sensitivity figure TSF characterizes an MRL by combining the sensitivities for multiple SOI models with the magnetic contrast:
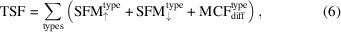
where ‘type’ stands for the types ‘normal’, ‘rough’ and ‘thin’ in Table 1 ▸. Together, these metrics provide a robust quantitative framework for evaluating the performance of the MRL in characterizing the SOI.

Thin films were deposited using ion-assisted direct-current magnetron sputter deposition within a high-vacuum environment, maintaining a background pressure of approximately 5.6 × 10^−5^ Pa. Single-crystalline Si substrates, measuring 20 × 20 × 0.5 mm and oriented along the 001 crystal axis, underwent thorough cleaning. The cleaning process involved successive ultrasonic baths using tri­chloro­ethyl­ene, acetone and propan-2-ol for 5 min each, followed by drying with nitro­gen gas. Argon gas (99.999% purity) was used as the sputtering medium at a pressure of 0.51 Pa (3.8 mTorr) and the substrates were maintained at room temperature. To enhance the attraction of low-energy ions during film growth, a bias voltage of −30 V was applied to the substrates and a substrate coil current of 5 A was utilized for the deposition of all samples.

In neutron reflectivity experiments on samples deposited on silicon substrates, the native SiO_2_ layer poses challenges due to potential chemical reactions or mixing with the sample, altering its properties. Additionally, the instability of SiO_2_ can lead to further oxidation or reactions over time, complicating reliable data acquisition. A stable capping layer is crucial to prevent such interactions and ensure accurate analysis. The chosen Al_2_O_3_ capping layer resists chemical alteration, minimizes substrate interactions and avoids phase shift concerns by maintaining a low SLD, preserving the sample’s integrity during measurements (Hegedus *et al.*, 2010 ▸; Kato *et al.*, 2009 ▸; Yeh *et al.*, 1993 ▸; Tonomura *et al.*, 2011 ▸; Kitano *et al.*, 2011 ▸; Wang *et al.*, 2021 ▸).

Continuous depositions from three magnetron sources for Co, Ti and Al were maintained, with material fluxes controlled via computer-regulated shutters. The three-inch sputtering targets for Co and Ti had purities of 99.5%, while the two-inch Al target also had a 99.5% purity. The magnetron discharges were operated by power-regulated supplies, where the ratio between Co and Ti was controlled by altering the power over the Ti target to achieve the desired composition. Low deposition rates were critical for ensuring precise control over layer thickness and roughness.

X-ray reflectivity measurements were carried out using a Malvern Panalytical Empyrean diffractometer equipped with Cu *K*α radiation and a PIXcel detector. For the incident beam, a Göbel mirror alongside a 0.5° divergence slit was utilized, while the diffracted beam optics featured a parallel beam collimator with a 0.27° collimator slit.

PNR experiments were conducted using the MORPHEUS instrument at the Paul Scherrer Institut (SINQ) in Switzerland. These experiments involved directing a polarized neutron beam onto the sample at small incidence angles (θ) to induce reflections at each interface, before detection by a ^3^He detector. PNR is particularly adept at detecting the spin-dependent SLD of the sample, which provides insights into its magnetization profile. The experiments produced two distinct reflectivity curves, corresponding to the two possible neutron spin states. Measurements were carried out with the samples positioned in an external magnetic field of approximately 20 mT and at angles ranging from 0° to 4° 2θ using a neutron wavelength of 4.825 Å.

## 3. Results and discussion

Using simulations and FOMs, the SOIs combined with the various MRLs were evaluated in terms of sensitivity over a scattering vector range.

**Figure 2:**
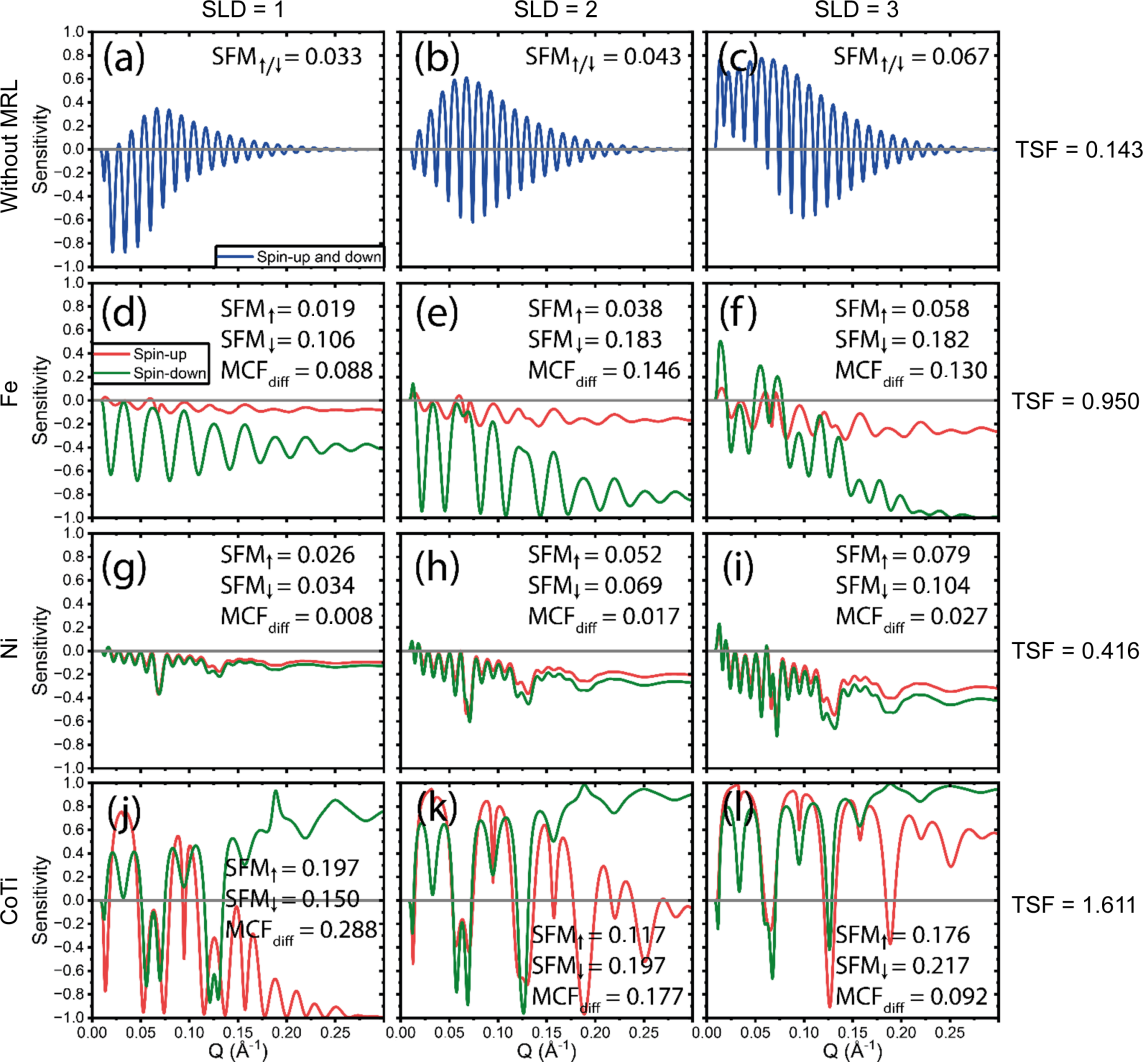
Sensitivity as a function of *Q* for ‘normal’ SOIs with SLDs of 1, 2 and 3 × 10^−6^ Å^−2^. (*a*)–(*c*) The sensitivity with no MRL. (*d*)–(*f*), (*g*)–(*i*) and (*j*)–(*l*) The Fe, Ni and CoTi MRLs, respectively. The total sensitivity TSF for each row is shown on the far right. Note that the plots with no MRL are calculated for either spin-up or spin-down and thus for non-polarized neutrons should be multiplied by a factor of 2 for comparison.

Fig. 2 ▸ illustrates the sensitivity as a function of *Q* for 12 distinct cases, all involving the ‘normal’ SOI. For cases without an MRL, shown in panels (*a*)–(*c*), PNR is unnecessary since there is no magnetic material present. Instead, NR alone suffices.

Figs. 2 ▸(*d*)–2(*f*) display the sensitivity for PNR experiments with an Fe MRL. The results demonstrate a clear separation between the two spin states, attributed to the high magnetic SLD of Fe. However, the separation is predominantly observed in the spin-down sensitivity curve, which shows significant dependence on the SOI, while the spin-up curve remains relatively close to zero as the reflectivity is dominated by the strong Fe reflectivity. Figs. 2 ▸(*g*)–2 ▸(*i*) show the results for an Ni MRL, where the magnetic SLD is lower than for Fe. While some separation between spin-up and spin-down states is observed, it occurs mainly at higher *Q* values and diminishes as the SLD of the SOI decreases.

Lastly, Figs. 2 ▸(*j*)–2 ▸(*l*) present the sensitivity for the novel Co_0.64_Ti_0.36_ MRL. These results exhibit a pronounced difference between spin-up and spin-down sensitivity curves, with overall sensitivity levels surpassing those of both Fe and Ni MRLs.

The visuals and the calculated TSFs for all cases clearly indicate that PNR with an MRL is significantly more effective than NR with no MRL. When comparing TSFs between the different MRLs, the results suggest that a higher magnetic SLD generally improves the separation between spin-up and spin-down states. However, despite Fe having a higher magnetic SLD than CoTi, the CoTi MRL demonstrates greater sensitivity for SOIs with SLDs of 1 and 2 × 10^−6^ Å^−2^. This implies that a lower overall SLD in the MRL, combined with a clear difference in SLD between spin-up and spin-down states, enhances sensitivity. Ultimately, the CoTi MRL achieves the highest TSF among all contenders, making it the most effective configuration for distinguishing spin states across the SOI range.

**Figure 3:**
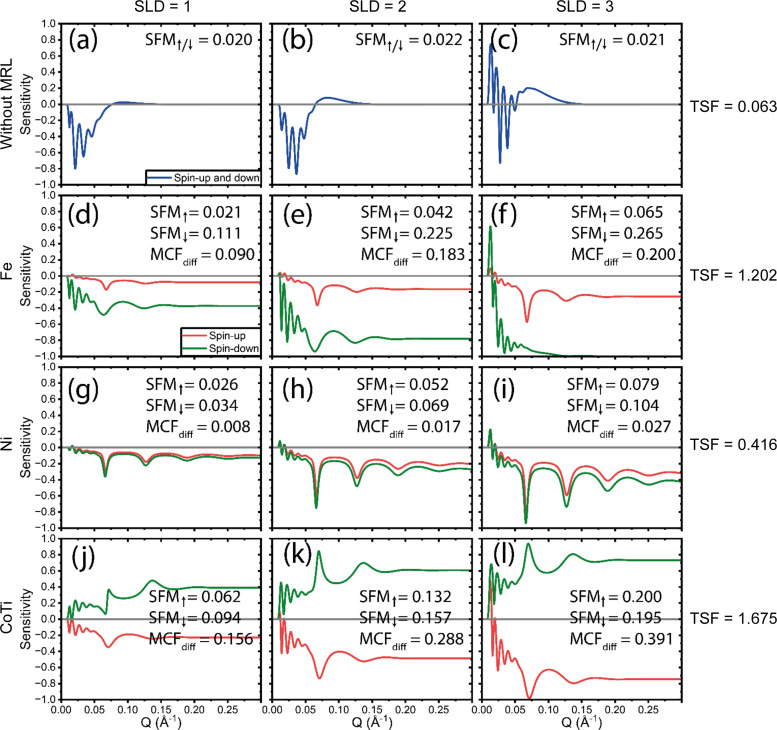
Sensitivity as a function of *Q* for ‘rough’ SOIs with SLDs of 1, 2 and 3 × 10^−6^ Å^−2^. (*a*)–(*c*) The sensitivity with no MRL. (*d*)–(*f*), (*g*)–(*i*) and (*j*)–(*l*) The Fe, Ni and CoTi MRLs, respectively. The total sensitivity TSF for each row is shown on the far right. Note that the plots with no MRL are calculated for either spin-up or spin-down and thus for non-polarized neutrons should be multiplied by a factor of 2 for comparison.

**Figure 4:**
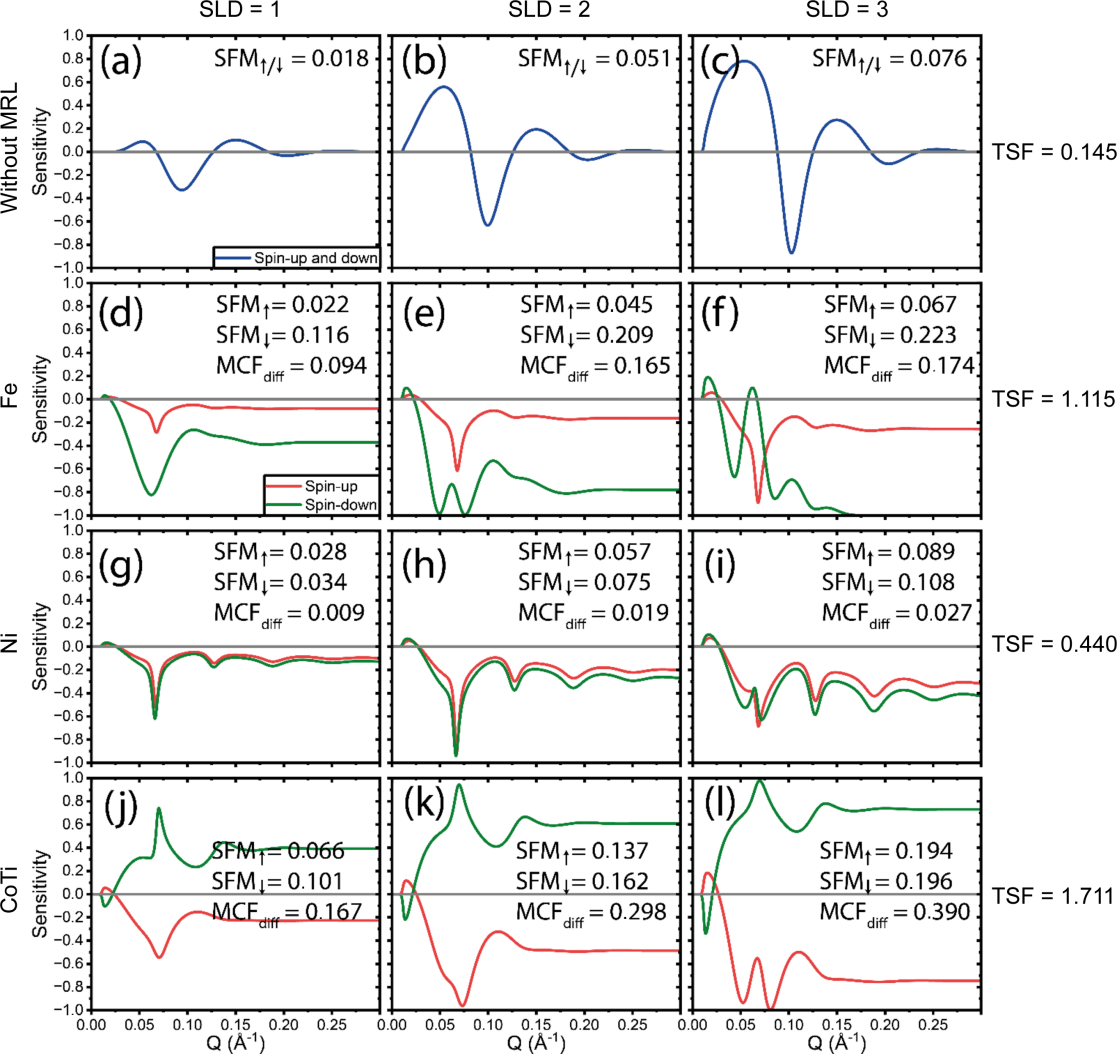
Sensitivity as a function of *Q* for ‘thin’ SOIs with SLDs of 1, 2 and 3 × 10^−6^ Å^−2^. (*a*)–(*c*) The sensitivity with no MRL. (*d*)–(*f*), (*g*)–(*i*) and (*j*)–(*l*) The Fe, Ni and CoTi MRLs, respectively. The total sensitivity TSF for each row is shown on the far right. Note that the plots with no MRL are calculated for either spin-up or spin-down and thus for non-polarized neutrons should be multiplied by a factor of 2 for comparison.

Comparing the ‘rough’ SOIs in Fig. 3 ▸ and ‘thin’ SOIs in Fig. 4 ▸ with Fig. 2 ▸, similar conclusions can be drawn. In this scenario, the superior performance of the CoTi MRL becomes even more pronounced versus the other MRLs. Additionally, it is evident that without an MRL the sensitivity is significantly diminished, with virtually no measurable sensitivity beyond *Q* = 0.13 Å^−1^. This highlights the critical role of incorporating an MRL in characterizing the details and roughness of the SOIs that manifest at higher reflection angles.

Other MRLs could be of interest to study and compare, but the key point is that a low nuclear and magnetic SLD MRL is needed for high sensitivity to low-SLD SOIs. Additional simulations using an FeCo MRL (Fig. S6) further support the claim that low spin-up and spin-down SLDs are critical for maximizing sensitivity to the SOI. Despite the reduced overall SLD compared with pure Fe, the FeCo MRL still dominates the spin-up reflectivity, confirming the importance of SLD matching between the MRL and SOI.

**Figure 5:**
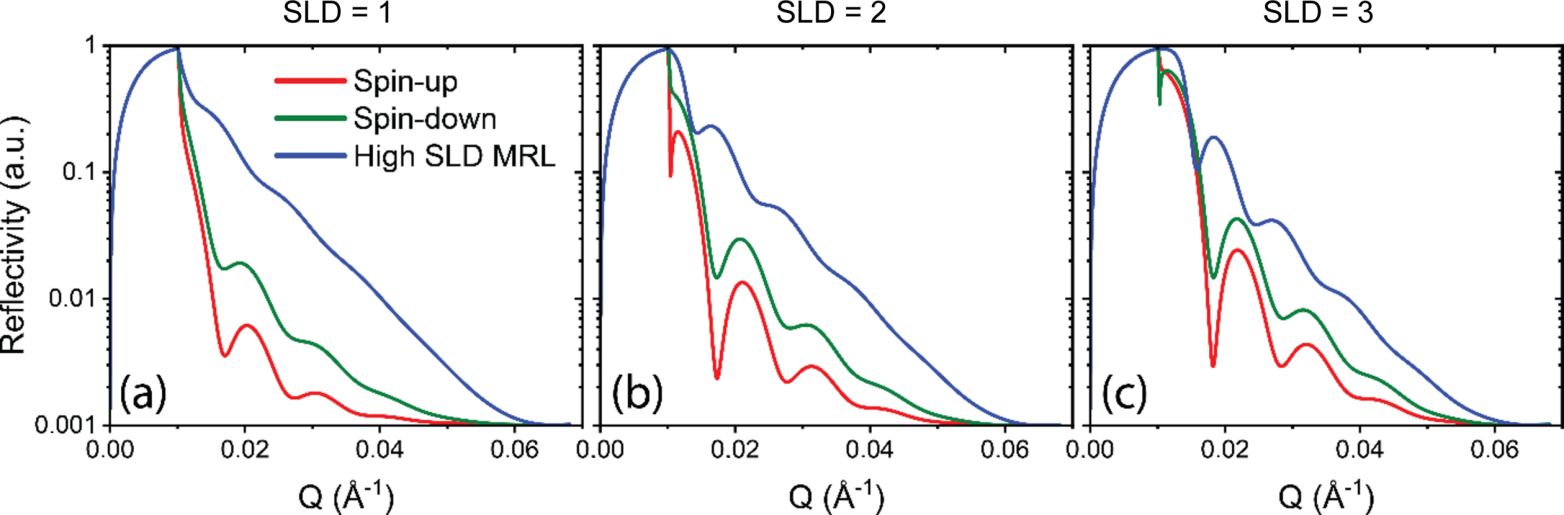
Polarized (red and green) and non-polarized (blue) NR simulations using a CoTi MRL (red and green) and a high-SLD non-magnetic MRL (blue) with SOI parameters of Λ = 500 Å and σ = 50 Å, *i.e.* ‘rough’. (*a*)–(*c*) Curves for an SOI SLD of 1, 2 and 3 × 10^−6^ Å^−2^, respectively. The high-SLD MRL has an SLD of 8 × 10^−6^ Å^−2^ and no magnetic SLD, thus no magnetic splitting.

Although CoTi is clearly a more effective MRL than Fe, since the strong scattering from Fe can overshadow or obscure contributions from the SOI, there are nevertheles situations where a high-SLD, even non-magnetic, MRL can enhance fringe visibility by lifting their intensity above background noise, as reported by Treece *et al.* (2019 ▸). As seen in the simulations in Fig. 5 ▸, a high-SLD MRL produces fringes with higher overall intensity than those obtained using the spin-dependent CoTi MRL. However, the differences in shape or features between the reflectivity curves for SOI SLD values of 1, 2 or 3 × 10^−6^ Å^−2^ become very small when a high-SLD MRL is used. This is because the MRL dominates the scattering, while the SOI, with much lower SLD values, contributes comparatively little. In contrast, with CoTi, which has lower spin-up and spin-down SLDs that are closer to those of the SOI, the reflectivity is more sensitive to variations in the SOI SLD. Therefore, the trade-off between achieving a higher signal and maintaining sensitivity to subtle differences comes down to the signal-to-noise ratio. Most NR experiments have reflectivity several orders of magnitude above the background. For example, as shown in Fig. 5 ▸, the signal near the critical edge is often four orders of magnitude higher than the background, indicating that using a high-SLD MRL to increase the signal may not be the most effective strategy when the goal is to extract detailed information from the SOI.

While the superior performance of CoTi is noteworthy on its own, its true strength lies in the tunability of its composition. By adjusting the ratio of Co to Ti during production, the nuclear SLD of the CoTi layer can be tailored within the range of −1.925 × 10^−6^ Å^−2^ (the value for Ti) to 2.265 × 10^−6^ Å^−2^ (the value for Co). Additionally, diluting the magnetic layer (Co) with a non-magnetic element like Ti reduces the magnetic density within the layer, thereby reducing the magnetic SLD. This allows the magnetic SLD to be tuned from 0 to 3.9 × 10^−6^ Å^−2^, offering remarkable flexibility in optimizing the CoTi MRL for specific experimental needs.

**Figure 6:**
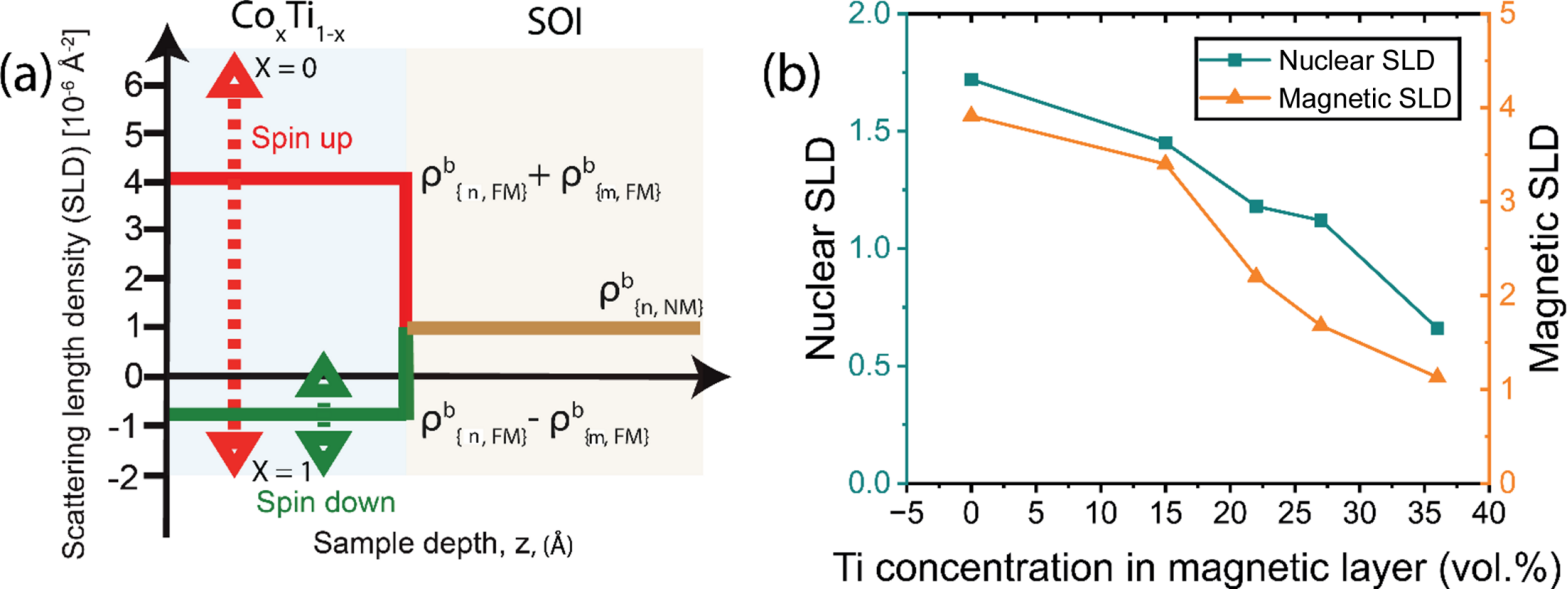
SLD tuning concept and fits on PNR measurements. (*a*) The theoretical SLD tuning concept, where the dashed red and green arrows show the tuning ranges for spin-up and spin-down, respectively. (*b*) The fitted nuclear (blue squares) and magnetic (orange triangles) SLDs for five samples with different volumetric ratios between Co and Ti.

The tunability range of the CoTi MRL is illustrated in Fig. 6 ▸(*a*). To validate the concept of SLD tunability, six CoTi MRL samples were fabricated, each capped with an Al_2_O_3_ layer. The corresponding measurements, fits and SLD profiles are provided in the supporting information. Fig. 6 ▸(*b*) plots the nuclear and magnetic SLD values as a function of Ti concentration in the CoTi MRL. As expected, both the nuclear and magnetic SLDs exhibit a clear decrease with increasing Ti concentration, confirming the tunability of the SLD. This capability of precisely optimizing the spin-up and spin-down SLDs within the MRL presents a powerful tool for achieving reliable and interpretable results from the SOI, making the CoTi MRL highly adaptable to a wide range of experimental conditions.

**Figure 7:**
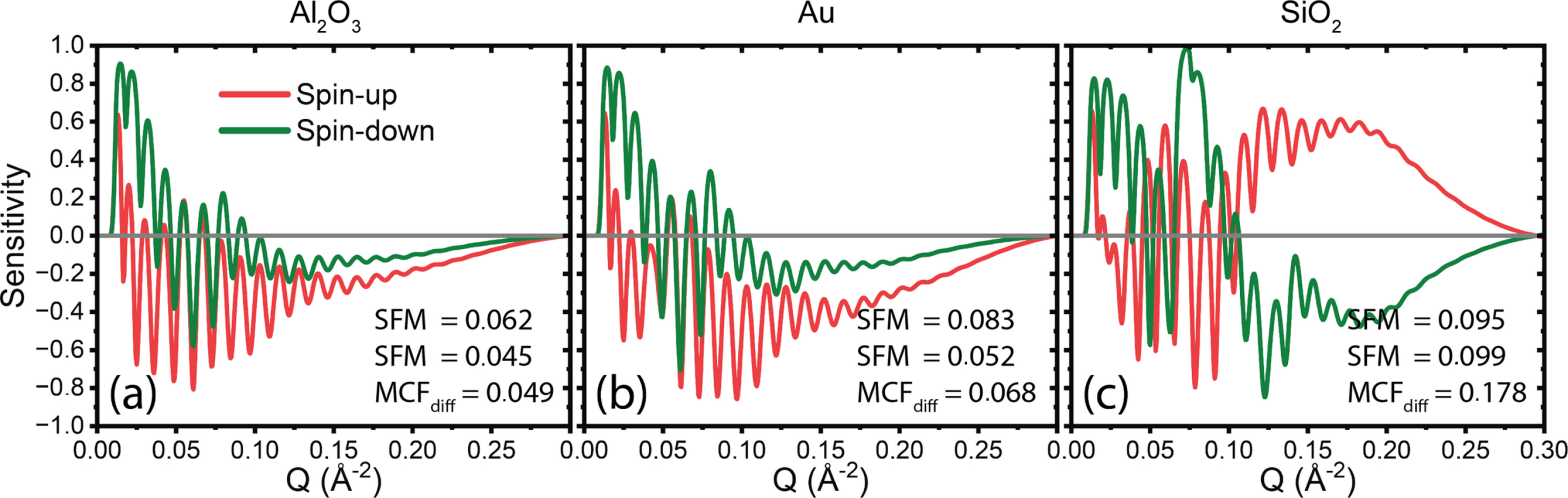
Sensitivity plots for a CoTi MRL with SOI parameters of Λ = 500 Å, σ = 15 Å and SLD = 3 × 10^−6^ Å^−2^, and three different capping layers, Al_2_O_3_, Au and SiO_2_. The capping layers all have the same layer thickness of 16 Å and an interface width of 8.6 Å.

A capping layer plays a crucial role in protecting the interface between the SOI and the MRL, preventing unwanted interactions or modifications. As previously discussed, the capping layer must be chemically stable, with common choices including Au, Al_2_O_3_ and SiO_2_. Among these, Au is the most chemically inert, offering excellent protection against oxidation and corrosion, though it has a significantly higher cost than the others. Al_2_O_3_ is also highly stable, both chemically and thermally, and provides a more cost-effective alternative. SiO_2_, while generally stable in dry environments, is less chemically inert, particularly under wet conditions where it may degrade. In addition to stability, the capping layer can influence the measurement sensitivity. As shown in the sensitivity plots in Fig. 7 ▸, SiO_2_ yields the most favourable sensitivity, probably due to its relatively low SLD. However, its lower chemical stability limits its suitability in reactive or humid environments. Al_2_O_3_ and Au show similar sensitivity performance, with Au performing slightly better. Still, the high cost of Au and its lower thermal and mechanical robustness make it less ideal for *in situ* studies involving elevated temperatures or pressures. Considering both sensitivity and material stability, Al_2_O_3_ presents the best overall compromise, offering excellent thermal, chemical and mechanical stability while maintaining good measurement sensitivity at a reasonable cost.

**Figure 8:**
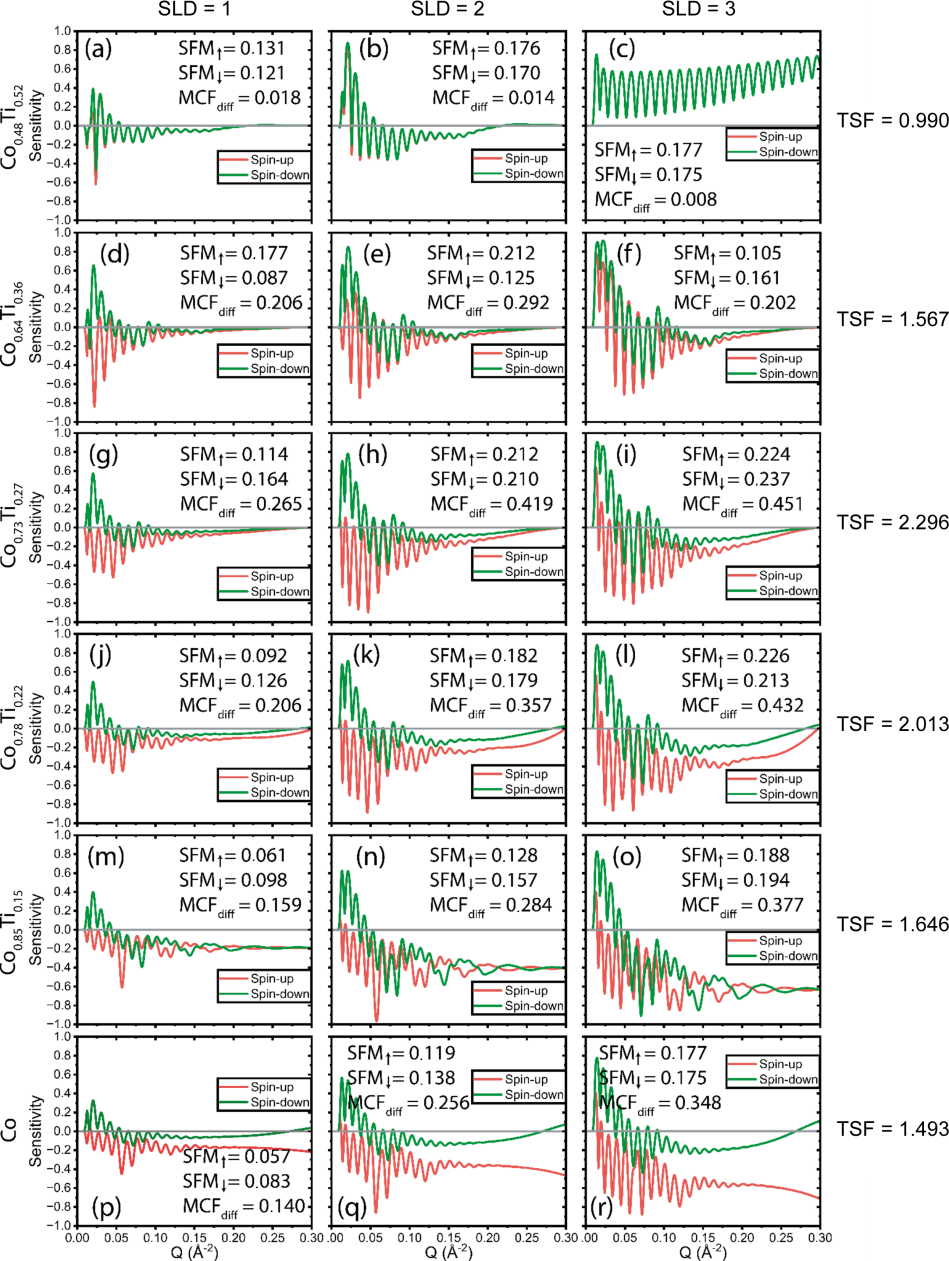
Sensitivity as a function of *Q* for ‘normal’ SOIs with SLDs of 1, 2 and 3 × 10^−6^ Å^−2^. (*a*)–(*c*), (*d*)–(*f*), (g)–(*i*), (*j*)–(*l*), (*m*)–(*o*) and (*p*)–(*r*) Sensitivity for six different CoTi MRLs, namely Co_0.48_Ti_0.52_, Co_0.64_Ti_0.36_, Co_0.73_Ti_0.27_, Co_0.78_Ti_0.22_, Co_0.85_Ti_0.15_ and Co, respectively. The total sensitivity TSF for each row is shown on the far right. All simulations include a capping layer of Al_2_O_3_ between the MRL and SOI.

Fig. 8 ▸ presents the sensitivity as a function of *Q* for normal SOIs with SLDs of 1, 2 and 3 × 10^−6^ Å^−2^ on six different CoTi MRLs, each with varying Co:Ti ratios. The results reveal that MCF_diff_ achieves its highest value for Co_0.73_Ti_0.27_ across all SOI SLDs, indicating that this ratio maximizes the difference between spin-up and spin-down states. However, when visually comparing the contrast between the spin states, the separation appears more pronounced at *Q* values above 0.15 Å^−1^ for MRLs with higher Co content, reaching a peak with pure Co which has the highest magnetic SLD. This tunability allows researchers to tailor the MRL to the specific *Q* range of interest for the SOI, ensuring optimal sensitivity for features of scientific importance. Among the MRLs tested, however, Co_0.73_Ti_0.27_ exhibits the highest TSF value, making it a reliable and versatile choice for experiments. As a result, Co_0.73_Ti_0.27_ emerges as a safe and effective option for most applications, balancing sensitivity and performance across varying conditions.

The importance of SLD tuning through compositional adjustment of the MRL is demonstrated in Fig. S7. For an SOI with SLD = 2 × 10^−6^ Å^−2^, Co_0.78_Ti_0.22_ offers the most favourable balance between contrast and magnetic splitting, highlighting how the Co:Ti ratio can be optimized to maximize information gain.

**Figure 9:**
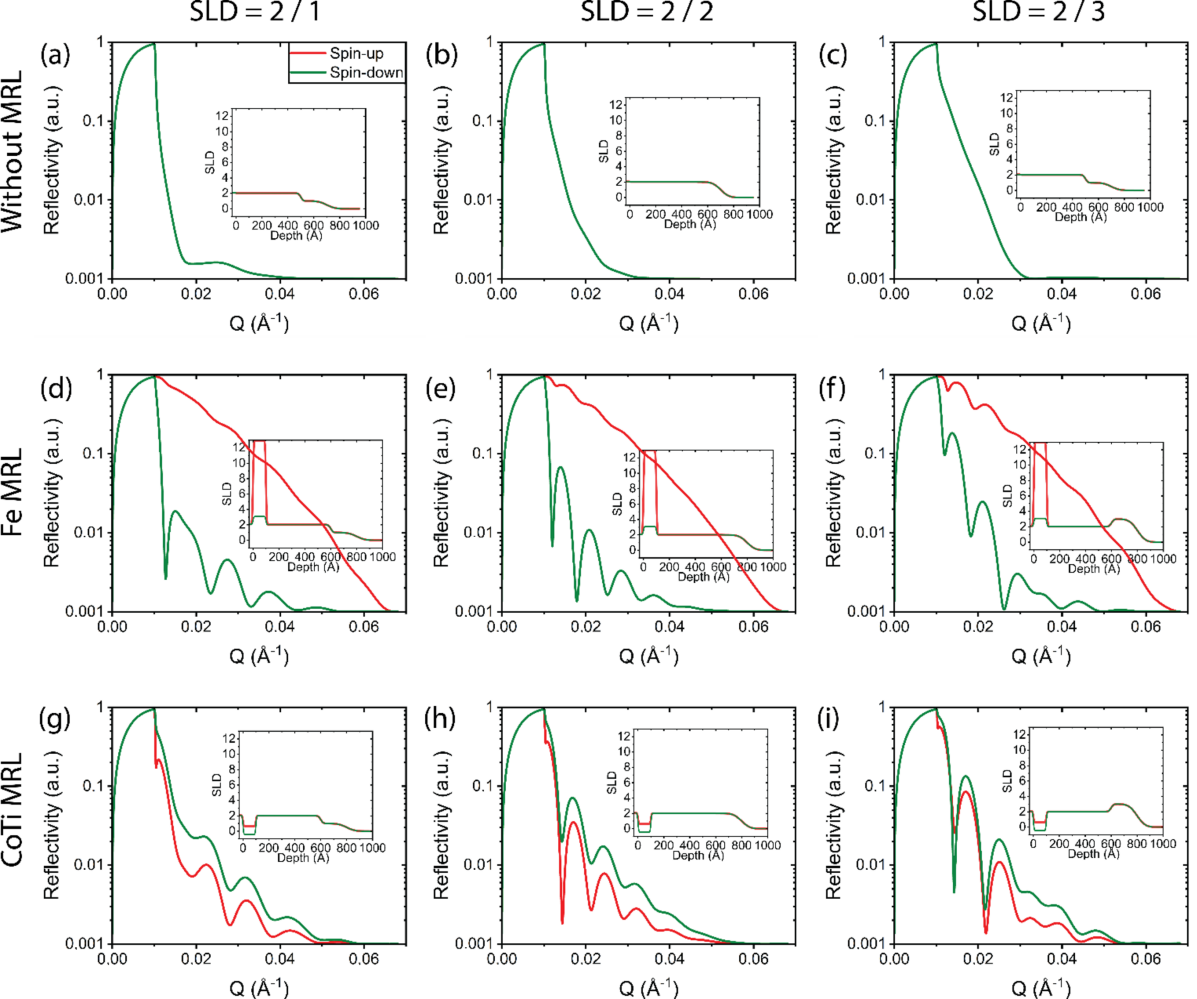
Polarized neutron reflectivity curves of two-layer SOI (‘complex’) models with parameters Λ_1_ = 500 Å and σ_1_ = 15 Å, and Λ_2_ = 200 Å and σ_2_ = 50 Å. (*a*)–(*c*) without an MRL, (*d*)–(*f*) with an Fe MRL and (*g*)–(*i*) with a CoTi MRL. Each panel shows the spin-dependent SLD profile in the inset. Panels (*a*), (*d*) and (*g*) show the scenario where the top part (Λ_2_) has an SLD of 1 × 10^−6^ Å^−2^, while (*b*), (*e*) and (*h*) show the scenario for an SLD of 2 × 10^−6^ Å^−2^ and (*c*), (*f*) and (*i*) show the scenario for an SLD of 3 × 10^−6^ Å^−2^.

Up to this point, comparisons between different MRLs have been made using a simplified model where the SOI consists of a single layer. However, real systems are typically far more complex (Ghoussoub *et al.*, 2018 ▸; Singh *et al.*, 2012 ▸; Beket *et al.*, 2024 ▸). In neutron reflectometry, single-layer systems are rarely the focus; instead, multilayers, diffusion profiles and interfacial phenomena are common. Conducting a fully comprehensive study where every possible SOI configuration is compared across all MRL and capping layer combinations would result in hundreds, if not thousands, of sensitivity plots. Our central argument is that a magnetic MRL with low spin-up and spin-down SLD provides the highest sensitivity to variations in the SOI, regardless of the SOI’s structural complexity. While this has been demonstrated using a single-layer SOI model, we now extend the analysis to a more complex two-layer SOI system, as illustrated in Fig. 9 ▸. When no MRL is used, it becomes difficult to distinguish characteristic differences for various SLDs within the upper part of the SOI layer (Λ_2_). In the case of the Fe MRL, the spin-down reflectivity curves vary between SOI configurations, but the spin-up curves are nearly identical due to Fe’s high SLD overwhelming the contrast. On the other hand, the CoTi MRL produces well defined fringes for both spin states and clear distinctions between different SOI configurations. These results confirm that the earlier conclusions still hold when moving from a single-layer to a two-layer SOI model. This suggests that CoTi remains the most effective MRL choice even as the complexity of the SOI increases, reinforcing its suitability for a broad range of real-world applications.

## 4. Conclusion

This study provides a systematic evaluation of optimal magnetic reference layers and introduces a versatile framework to enhance sensitivity for studying a wide range of materials, especially soft matter, using neutron reflectometry. CoTi-based MRLs were shown to outperform Fe and Ni MRLs significantly, as well as the absence of an MRL, in enhancing the sensitivity of neutron reflectometry.

Across a matrix of nine samples of interest varying in thickness, roughness and scattering length density (SLDs of 1, 2 and 3 × 10^−6^ Å^−2^), CoTi consistently achieved the highest spin contrast (MCF_diff_) and total sensitivity (TSF).

The tunability of CoTi, enabled by adjusting the Co:Ti ratio, allows for tailored nuclear and magnetic SLDs, with Co_0.73_Ti_0.27_ identified as the optimal composition. This versatility ensures superior performance across diverse SOIs and *Q* ranges, establishing CoTi as a robust and flexible choice for PNR experiments, particularly in soft-matter and thin-film research.

The ability to tailor CoTi MRLs for specific experimental requirements, combined with the open-source availability of our simulation framework, ensures that this approach can be readily adopted and further developed by the scientific community. These advances have the potential to broaden the applicability of reflectometry in exploring novel materials, complex interfaces and thin-film systems with higher sensitivity and accuracy.

## Supplementary Material

Additional details of simulations and figures. DOI: 10.1107/S1600576725004674/ei5132sup1.pdf

## Figures and Tables

**Table 1:** Different samples of interest and their SLD ρ, thickness Λ and surface roughness σ

Type	SOI SLD (× 10^−6^ Å^−2^)	Thickness Λ (Å)	Roughness σ (Å)
Normal	1	500	15
Normal	2	500	15
Normal	3	500	15
Rough	1	500	50
Rough	2	500	50
Rough	3	500	50
Thin	1	50	15
Thin	2	50	15
Thin	3	50	15
Complex	1	500 / 200	15 / 50
Complex	2	500 / 200	15 / 50
Complex	3	500 / 200	15 / 50

**Table 2:** Different magnetic reference layers and their nuclear and magnetic SLDs ρ All MRLs have a thickness Λ = 100 Å and surface roughness σ = 5 Å.

MRL	ρ_n_ (× 10^−6^ Å^−2^)	ρ_m_ (× 10^−6^ Å^−2^)
Fe	8	5
Ni	9.4	1
CoTi	1	2
