# Supplementary material for: Optimization of magnetic reference layer for neutron reflectometry
Source: J Appl Crystallogr. 2025 Jul 22;58(Pt 4):1299–310. doi: 10.1107/S1600576725004674 (PMC12321035; doi:10.1107/S1600576725004674)
Supplement: Supplementary file 1 [file j-58-01299-sup1.pdf]

JOURNAL OF APPLIED CRYSTALLOGRAPHY

**Volume 58 (2025)**

**Supporting information for article:**

# Optimization of magnetic reference layer for neutron reflectometry

**Anton Zubayer, Fredrik Eriksson, Naureen Ghafoor, Jochen Stahn, Jens Birch and Artur Glavic**

This document provides simulation details and all relevant X-ray and neutron reflectivity curves.

The sensitivity curves presented in this study were simulated using GenX 3, a widely used software for reflectometry data fitting and model-based simulations. GenX leverages the differential evolution algorithm to optimize model parameters by minimizing the difference between experimental data and simulated results. A custom Python script was developed within GenX to calculate reflectivity and sensitivity curves for various magnetic reference layers (MRLs) and samples of interest (SOIs).

Each layer in the multilayer structure was defined as a 'Layer' object, specifying properties such as density, thickness Λ, and roughness σ. The sample stack was then assembled as a 'Stack' object, enabling repetition of specific multilayer units. For instance, the CoTi layers were modelled with adjustable Co-to-Ti ratios, providing tunability of both nuclear and magnetic SLDs. The script simulated reflectivity for spin-up R↑↑(Q) and spin-down R↓↓(Q) polarization states, and the sensitivity was calculated as the integrated area between the curves and reference line at Y = 0 across a range of momentum transfer (Q) values.

To streamline the calculations, the script featured modular functions for simulating specific datasets under different polarization conditions (e.g., spin-up or spin-down). We incorporated models such as 'normal,' 'rough,' and 'thin' SOI configurations, as well as SOIs with varying SLDs (1, 2, and $3\times10^{-6}$ Å$^{-2}$), however the reader can adjust the parameters as desired.

The script enabled systematic exploration of MRL configurations, in our case including Fe-, Ni-, and CoTi-based layers, as well as the absence of an MRL. The tunability of the CoTi MRL was particularly highlighted, as the script allowed for stepwise variation of Co-to-Ti ratios, generating nuclear SLDs ranging from −1.925 (Ti) to 2.265 (Co) [$10^{-6}$ Å$^{-2}$], and magnetic SLDs ranging from 0 to 3.93 [$10^{-6}$ Å$^{-2}$]. Reflectivity simulations were run for a range of CoTi compositions, and the results were validated against experimental SLD profiles.

The sensitivity calculations were performed using a custom Python script implemented within GenX 3, leveraging its modular framework for neutron reflectometry simulations. The sample is initialized as a 'Stack' object, combining layers with specified physical and magnetic properties. For example, the CoTi layer represents the magnetic reference layer (MRL), while the GasLayer acts as the SOI. These layers are combined into a stack and subsequently into a 'Sample' object that includes the ambient and substrate conditions:

```
ML = model.Stack(Layers=[CoTi, GasLayer], Repetitions=1)
sample = model.Sample(Stacks=[ML], Ambient=Amb, Substrate=Sub)
```

Reflectivity simulations are carried out using six datasets, corresponding to different polarization states and configurations of the multilayer. For each dataset, the SimSpecular method is used to compute the reflectivity for a given momentum transfer (Q) range, while the SimSLD method calculates the scattering length density (SLD) profile:

```
inst.setPol('uu')  # Spin-up polarization
d = data[0]        # First dataset
I.append(sample.SimSpecular(d.x, inst))  # Compute reflectivity
if _sim:
    SLD.append(sample.SimSLD(None, None, inst))  # Compute SLD profile
```

This approach is repeated for spin-down polarization ('dd'), enabling the calculation of spin-dependent reflectivity. The sensitivity curves are derived by toggling the sample structure between different configurations (e.g., with or without the gas layer or magnetic reference layer) and comparing the reflectivity results. For example:

- **Datasets 0 and 1**: Full sample configuration (CoTi + GasLayer) with spin-up (uu) and spin-down (dd) polarizations.
- **Datasets 2 and 3**: Simplified configuration (CoTi only) for spin-up and spin-down polarizations.

By comparing the full and simplified configurations, the script evaluates the sensitivity of the reflectivity signal to changes in the sample structure.

The **spin sensitivity**, is calculated for datasets 4 and 5 by combining the results from earlier datasets. The spin-up sensitivity (I[4]) and spin-down sensitivity (I[5]) are computed as follows:

```
I[4] = (I[0] - I[2]) / (I[0] + I[2])  # Spin-up sensitivity
I[5] = (I[1] - I[3]) / (I[1] + I[3])  # Spin-down sensitivity
```

These calculations quantify the sensitivity of the MRL by measuring the contrast between the reflectivity signals of the full and simplified configurations. This methodology allows for a systematic comparison of different MRLs (e.g., Fe, Ni, CoTi, or no MRL) under various SOI conditions. Furthermore, it provides a framework to analyse the tunability of CoTi MRLs by varying Co-to-Ti ratios, enabling precise adjustments to the nuclear and magnetic SLDs.

The full code is available at: https://github.com/Azubayer/GenX-sensitivity

MATLAB scripts were used to analyse sensitivity curves and compute figures of merit (FOMs) for assessing the performance of magnetic reference layers (MRLs) under various sample conditions. Key calculations include:

- **Sensitivity for spin-up and spin-down:** The absolute area under the sensitivity curves for spin-up and spin-down polarizations was calculated to evaluate their individual contributions to the overall sensitivity.
- **Sensitivity difference**: The integral of the absolute difference between spin-up and spin-down sensitivity curves across the Q-range was computed, providing a measure of magnetic contrast between the two polarization states.
- **Combined FOM**: The figures of merit for spin-up, spin-down, and sensitivity difference were combined to provide a comprehensive metric for comparing the effectiveness of different MRLs.

These simulations provided a robust and reproducible framework for evaluating MRL performance, enabling the identification of optimal configurations for polarized neutron reflectometry.

The full code is available at:

https://github.com/Azubayer/GenX-sensitivity

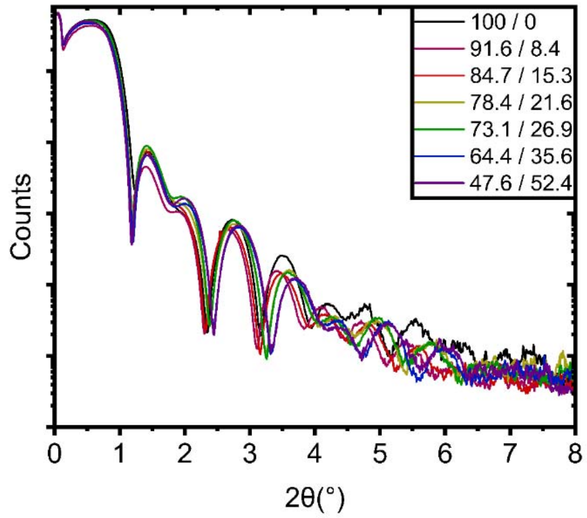


Figure S1. XRR measurements of 7 CoTi MRL samples with varying ratios between Co and Ti, all with $Al_2O_3$ capping layer.

Figure S1 shows that the layer thicknesses are approximately the same. Thereby confirming that the sensitivity of the different ratios of Co and Ti is not due to thickness differences, but solely due to composition.

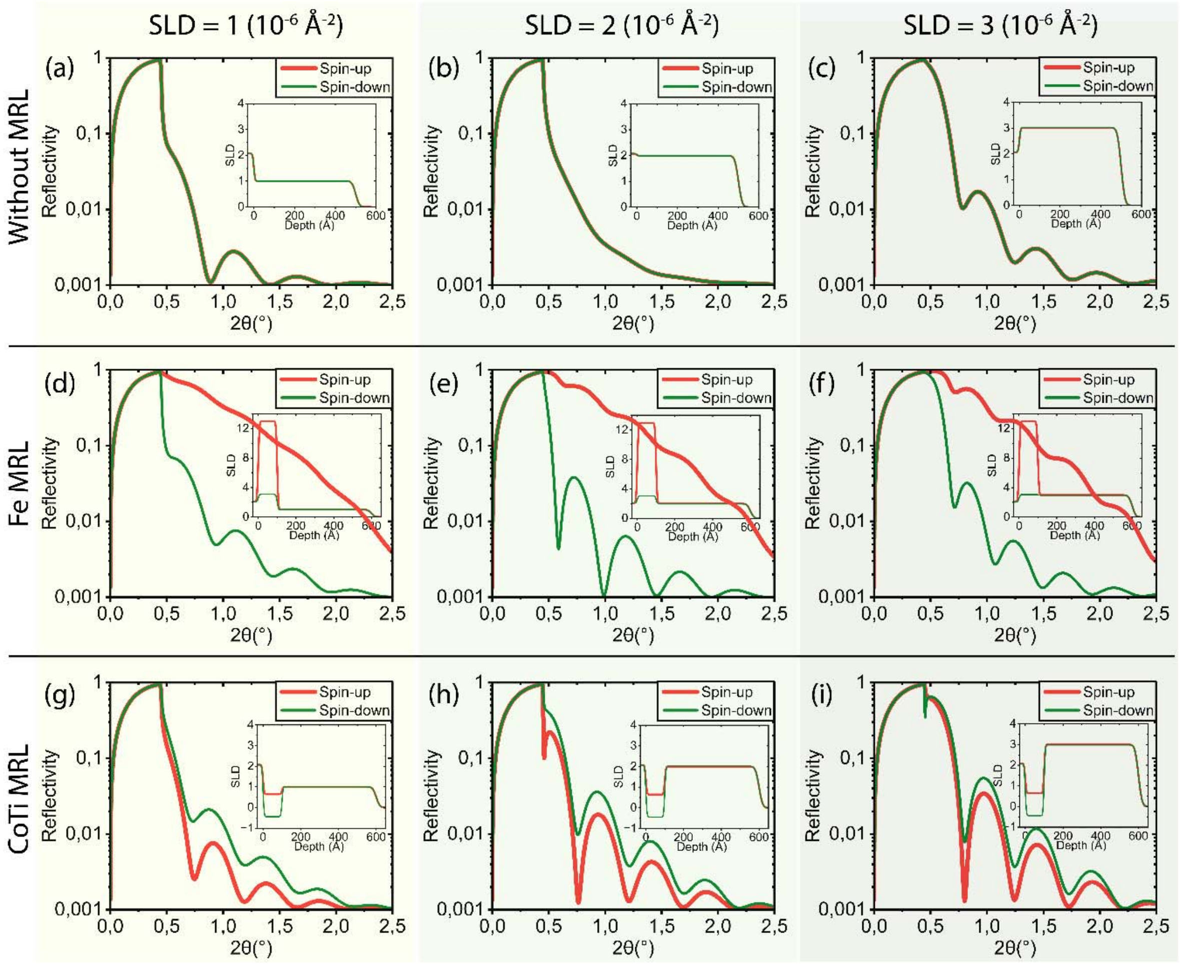


Figure S2. Polarized neutron reflectivity simulations on SOI. (a-i) corresponds to the type “normal” from Table 1. The inset in each graph shows the SLD depth profile. (a-c) are samples without MRL but with an SOI SLD of 1, 2 and 3 ($10^{-6}$ Å$^{-2}$) respectively. (d-f) are samples with Fe MRL but with an SOI SLD of 1, 2 and 3 ($10^{-6}$ Å$^{-2}$) respectively. (g-i) are samples with $Co_{64}Ti_{36}$ MRL but with an SOI SLD of 1, 2 and 3 ($10^{-6}$ Å$^{-2}$) respectively.

Figure S2 shows that visually, the reflectivity curves have more pronounced features of the SOI when the CoTi MRL was used compared to Fe or when no MRL was used.

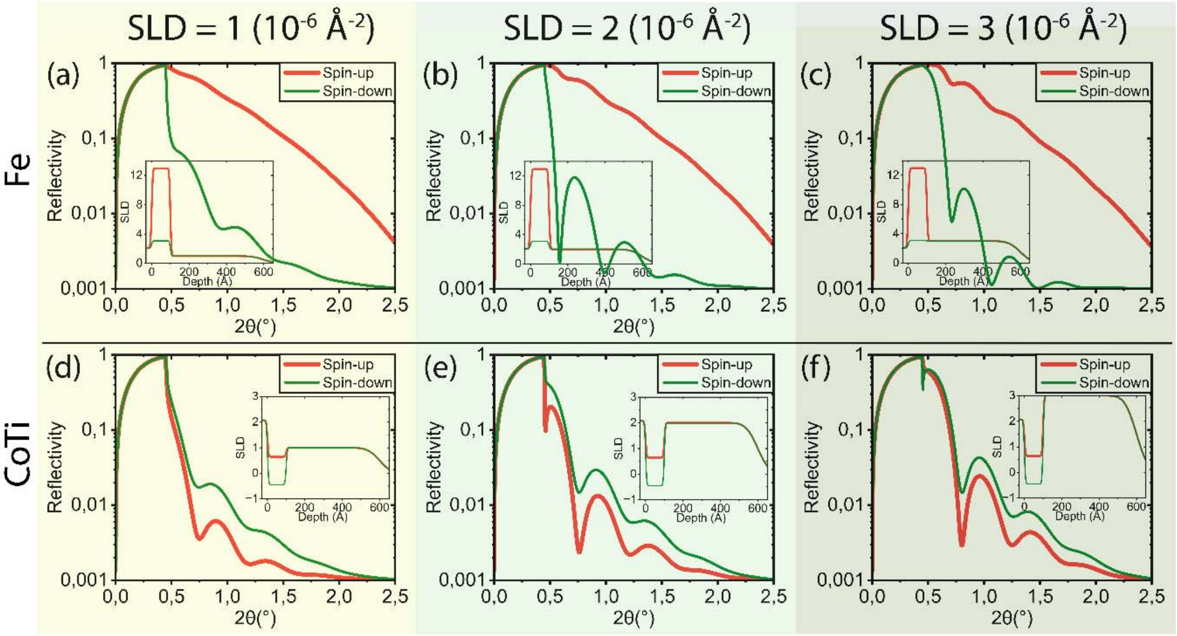


Figure S3. Polarized neutron reflectivity simulations on 50 Å rough SOI on top of two different MRLs. Top row for Fe and bottom row for CoTi. Each column represents an SOI SLD of 1, 2 and 3 ($10^{-6}$ Å$^{-2}$). All samples are related to type "rough" from Table 1.

Figure S3 shows that visually, also here, the reflectivity curves have more pronounced features of the SOI when the CoTi MRL was used compared to when Fe was used. Thus both for type "normal" and "rough" the CoTi MRL is preferred.

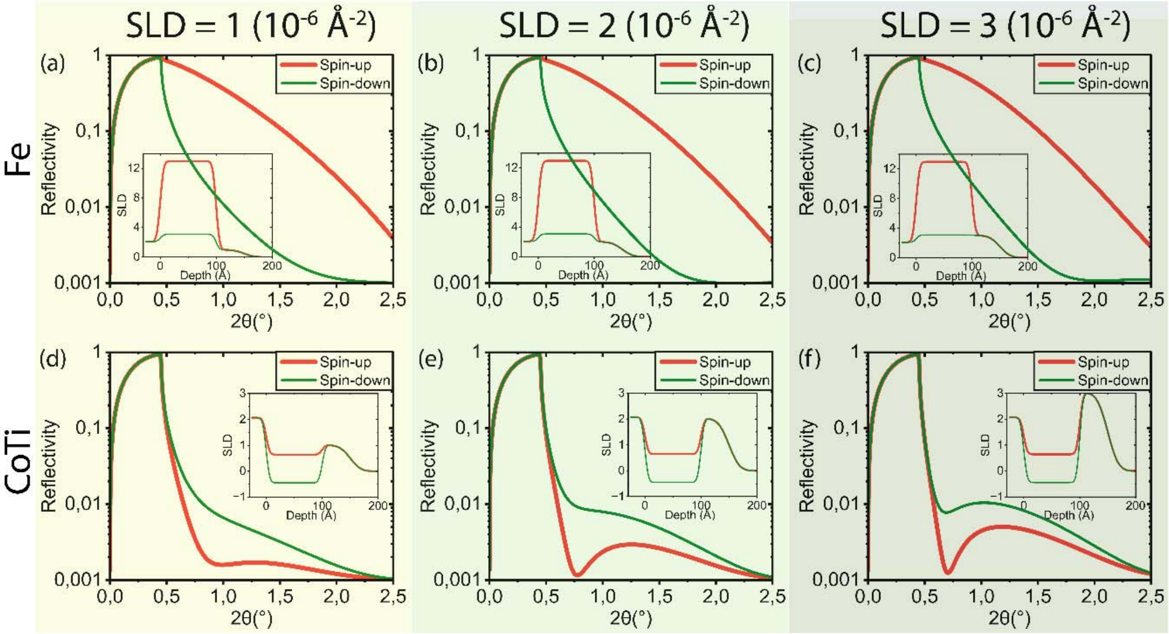


Figure S4. Polarized neutron reflectivity simulations on 50 Å thin SOI layers on top of two different MRLs. Top row for Fe (Samples 16-18) and bottom row for CoTi (Samples 19-21). Each column represents an SOI SLD of 1, 2 and 3 ($10^{-6}$ Å$^{-2}$). All samples are related to type “thin” from Table 1.

Figure S4 shows that visually, for a third time, the reflectivity curves have more pronounced features of the SOI when the CoTi MRL was used compared to when Fe was used. Thus, all three types “normal”, “rough” and “thin” the CoTi MRL is preferred, establishing its superiority.

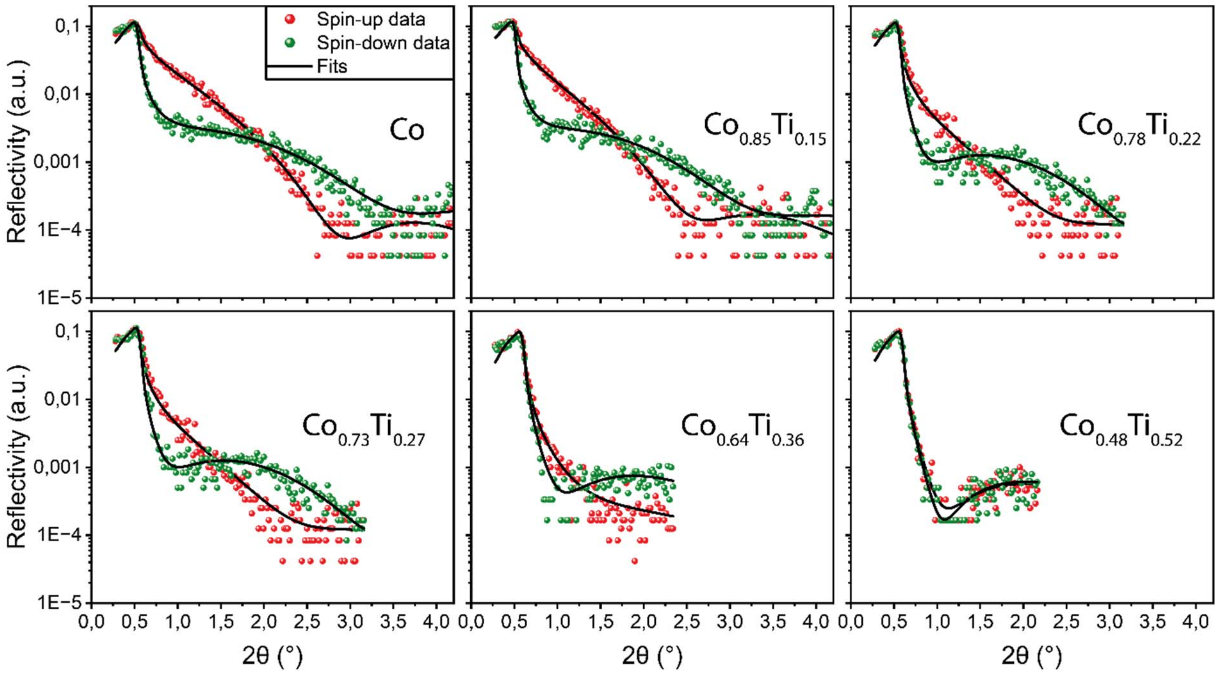


Figure S5. Polarized neutron reflectivity measurements and fits of 6 samples of $Co_xTi_{1-x}$ MRL with $Al_2O_3$ capping layer with SOI on top for three different SLDs; 1, 2 and 3 ($10^{-6}$ Å$^{-2}$). All samples have varying amounts of Co to Ti ratio.

Figure S5 shows the data and fits of the 6 different CoTi samples with varying amounts of Co to Ti ratio. These fits resulted in the values of nuclear and magnetic SLD seen in Figure 5.

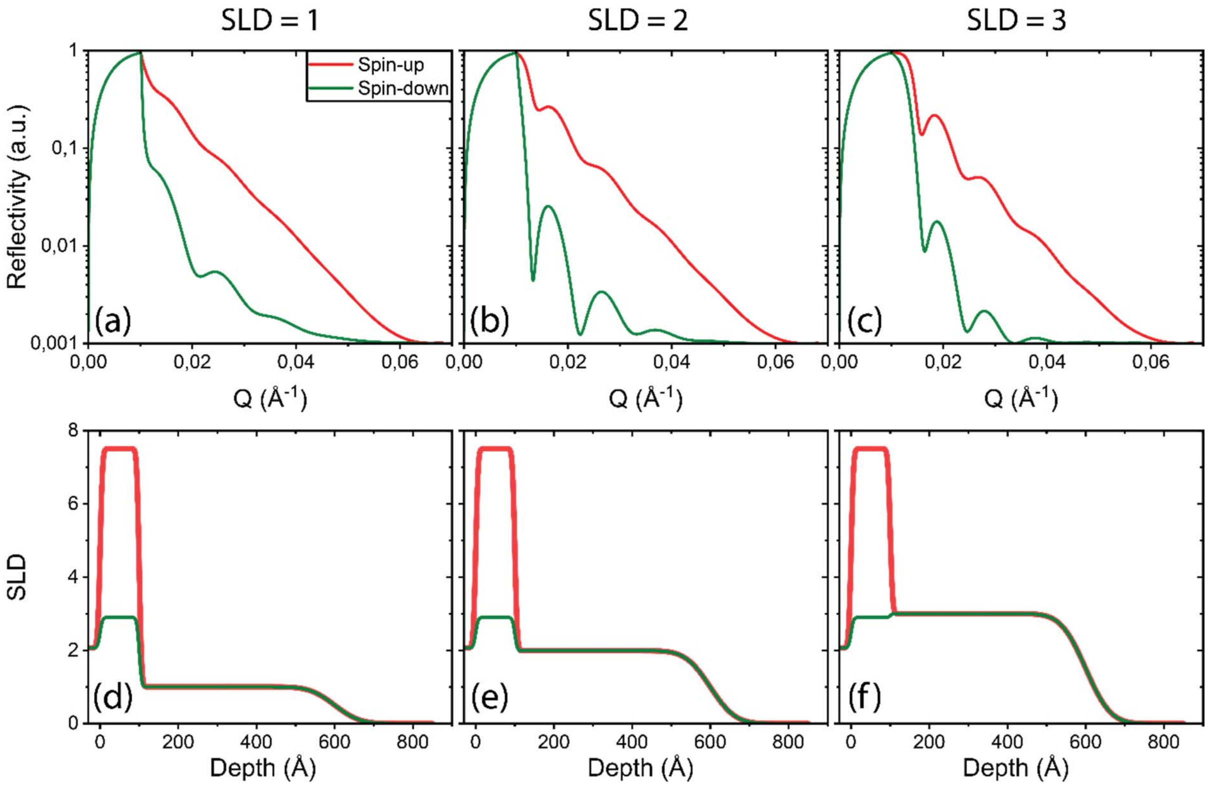


Figure S6. Polarize neutron reflectivity curves (a-c) with corresponding spin-dependent SLD depth-profiles (d-f) using an FeCo MRL. The SOI varied from 1, 2 and 3 ($10^{-6}$ Å$^{-2}$).

As shown in Figure S6, although the inclusion of Co reduces the overall SLD of the MRL compared to a pure Fe MRL, the spin-up reflectivity curves are still heavily dominated by the MRL's intensity. In other words, the MRL continues to overpower the spin-up reflectivity signal, much like in the case of the pure Fe MRL. Additionally, the greatest sensitivity to changes in the SOI is observed when the SOI SLD is 3 ($10^{-6}$ Å$^{-2}$), compared to values of 2 or 1. This reinforces the conclusion that, for maximum information gain, the spin-up and spin-down SLDs of the MRL should be low and closely matched to the SLD of the SOI.

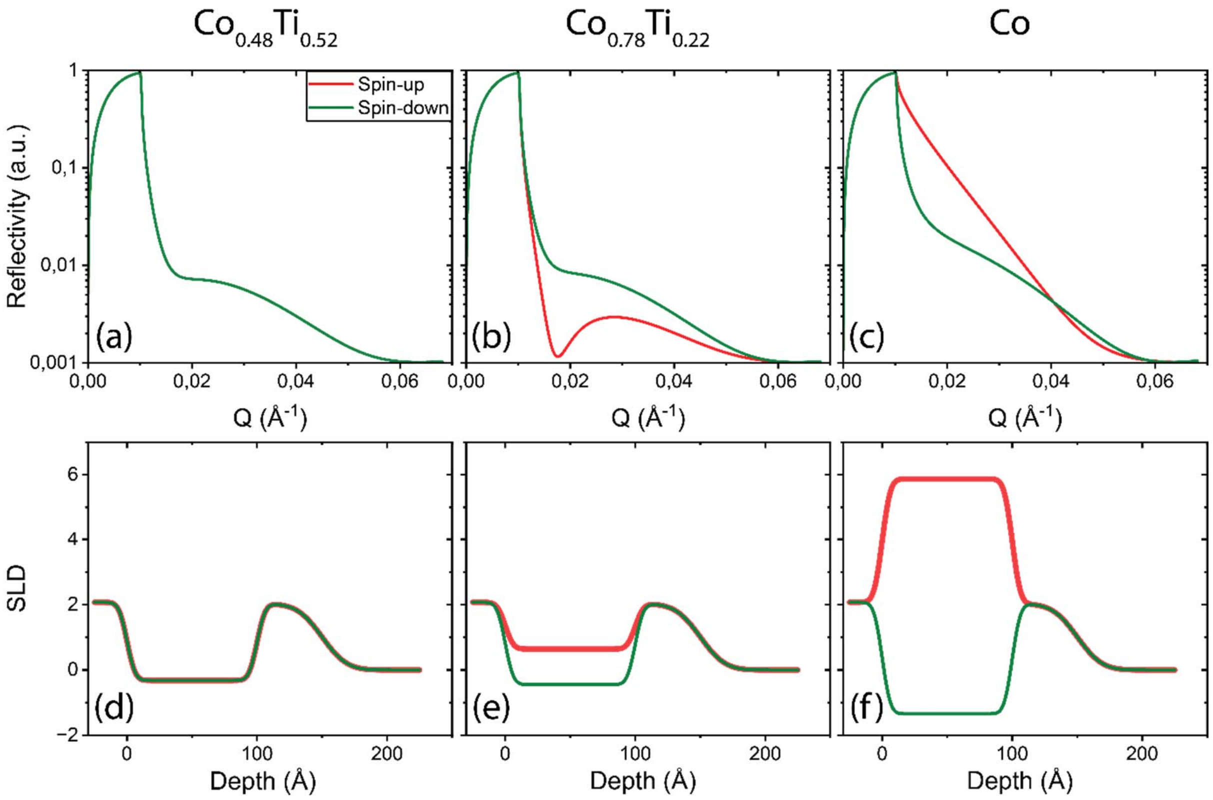


Figure S7. Polarized neutron reflectivity curves (a-c) and corresponding spin-dependent SLD depth-profiles (d-f). (a, d) belongs to Co0.48Ti0.52, (b, e) to Co0.78Ti0.22 and (c, f) to Co. All three samples have an SOI of SLD = 2 ($10^{-6}$ Å$^{-2}$), 2 Λ = 50 Å and σ = 15 Å ("thin").

Figure S7 demonstrates the importance of SLD tuning of the MRL in relation to the SLD of the SOI. When the magnetic SLD is too small or nearly absent, as in $Co_{0.48}Ti_{0.52}$, there is no observable magnetic splitting and therefore no added benefit from spin polarization. In contrast, for pure Co, the spin-up and spin-down SLDs of the MRL are both highly mismatched with the SOI SLD, resulting in strong scattering but no visible fringe features due to the excessive contrast. The intermediate composition, $Co_{0.78}Ti_{0.22}$, provides the most information: both spin channels exhibit distinct fringes and moderate magnetic splitting, offering optimal sensitivity without overwhelming the signal. This indicates that, for an SOI with an SLD of 2 ($10^{-6}$ Å$^{-2}$), $Co_{0.78}Ti_{0.22}$ is the most effective MRL, balancing contrast and magnetic sensitivity. The result highlights the value of tuning the MRL SLD through careful adjustment of the Co-to-Ti ratio.
